# Reduction in distraction due to perceptual load: A failure to replicate

**DOI:** 10.1177/03010066251364203

**Published:** 2025-08-06

**Authors:** Robert J. Snowden, Nicola S. Gray

**Affiliations:** 2112 Cardiff University, UK; 7759 Swansea University, UK; 7757 Swansea Bay University Health Board, UK

**Keywords:** attentional capture, divided attention/resource competition, visual search, attention

## Abstract

Perceptual load theory states that there are limited processing resources, but that these must always be fully employed. It has been used to predict and explain the commonly reported finding that irrelevant distractors influence behaviour when the task has low load (such as processing just one target element) but not when the task has high load (processing many target elements). We attempted to replicate this effect over a series of six experiments that manipulated the location of the distractor, the duration of the display, and different levels of load. We examined both the distracting effects caused by a “neutral” distractor, and response-biases (congruence effects) that occur when the distractor is either congruent or incongruent with the target. Strong distraction and congruence effects were found with central distractors and weaker effects were found with peripheral distractors. These effects appeared to be independent of the level of perceptual load in all conditions. Our findings thus do not support the tenants of perceptual load theory and fail to replicate the many findings that do support this theory.

## Introduction

As stated by James (1890), attention involves “*withdrawing from some things in order to deal effectively with others*.” Thus, when dealing with an everyday image containing many objects, attention allows us to select one of these for deeper processing while ignoring the other objects. However, the mechanism by which this is achieved, and the “fate” of these unattended objects has remained a central question in perception and cognition for over a century (Driver, 2001).

Perceptual load theory has been a major driver in advancing thinking about this issue (Lavie, 1996, 2010; Lavie & Tsal, 1994). Perceptual load theory claims that attention has a limited capacity but that it is always fully employed (i.e., the person cannot decide to only use part of these processing resources). In conditions where there is a clear distinction between which information needs to be processed (the relevant stimuli) and which are not (the irrelevant stimuli), attention is first applied to the relevant stimuli. Under conditions of “high load,” for instance when there are many relevant stimuli to be processed, attentional capacity will be exhausted by the relevant items and so the irrelevant items will not be processed. However, under conditions of “low load,” for instance when only a single relevant item is presented, attentional capacity will be only minimally used leaving capacity for the irrelevant item(s) to be processed.

**Figure 1.**
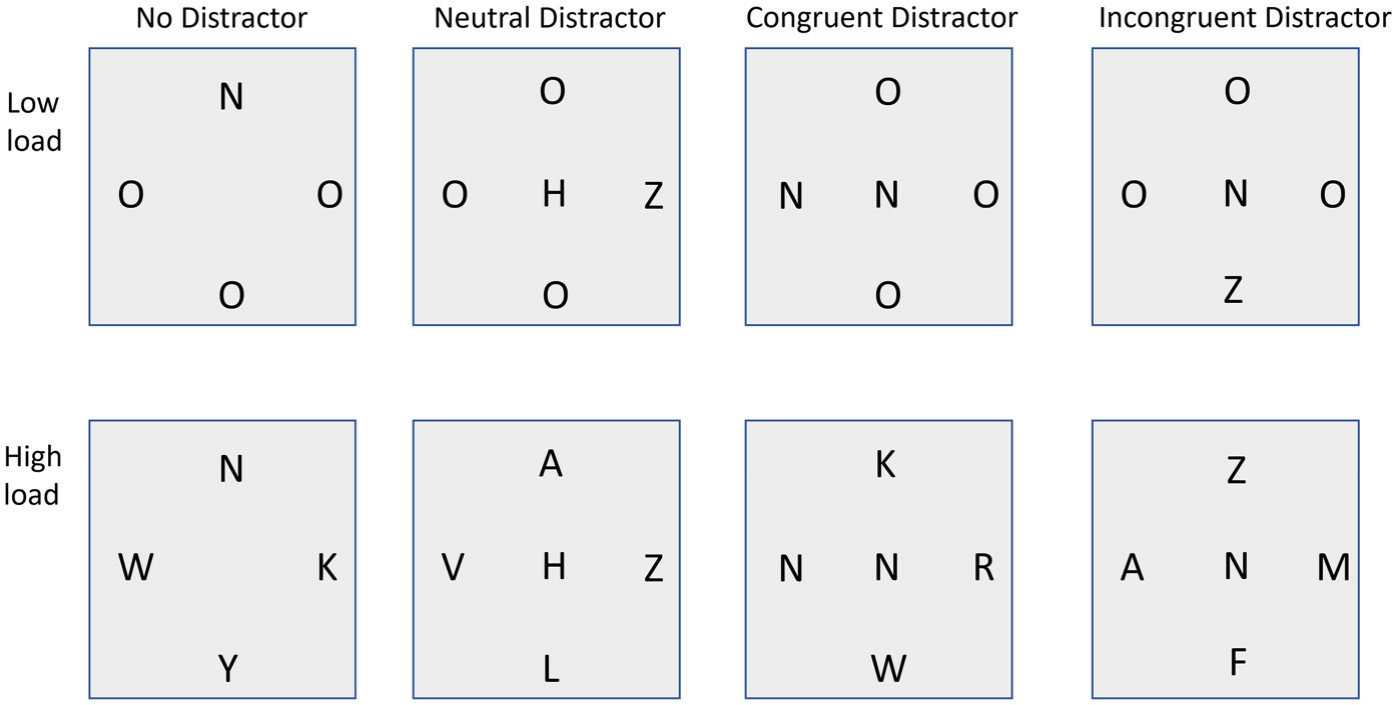
Illustration of the displays used in Experiments 1 and 2.

Many studies have supported this idea (Murphy et al., 2016). The main paradigm employed is illustrated in Figure 1. Here several “relevant” items are presented within a circular array, with one of the items being a “target” item. The task is to decide if the target item is one of two possible letters (e.g., Z or N). At the same time, a single “distractor” letter is presented away from the circular array (normally to one side) and the participant is fully aware that this item is irrelevant and should be ignored. The distractor letter is either the same letter as the target (e.g., a Z when the target is a Z, termed “congruent”), or the other possible target letter (e.g., an N when the target is a Z, termed “incongruent”). Under conditions of low load (where other relevant letters are very different to the target letter, or even absent) the “typical” result is that people are fast for the congruent trials, but slow for the incongruent trials. This pattern shows that the participants must have processed the distractor letter and that this letter has produced a response-interference effect. Under conditions of high load (where the other relevant letters share similarities with the target letter) response times are much slower reflecting the difficulty in finding the target letter. However, the “classic” result (Lavie & Tsal, 1994) is that response times for the congruent and incongruent trials are similar. This suggests that either, (a) the distractor image was not processed, or (b) the distractor was processed but was no longer able to produce response interference. The former explanation is usually accepted.

Despite the success of load theory, there have been some criticisms of the concept (Benoni & Tsal, 2013; Giesbrecht et al., 2014; Khetrapal, 2010) and some studies that produce findings that run counter to the idea of load theory. For instance, some versions of low load fail to produce distraction effects (Eltiti et al., 2005; Johnson et al., 2002; Tsal & Benoni, 2010). Many of these effects, including the “standard” finding of reduced/abolished distraction at high load, can also be explained by other models, such as those involving salience-based accounts (Neokleous et al., 2016) or involving modelling of competition for resources (Scalf et al., 2013).

These empirical studies and computational modelling all agree with the standard result that increased load reduces/abolishes the effects of distraction in most circumstances. In this article, we would like to present results from a series of experiments that fail to replicate this basic finding despite extensive attempts to find a condition in which it occurred. Our initial interest in completing these experiments came from other studies where we had attempted to look at whether distraction by “emotional” images would be spared in comparison to non-emotional distractors even in conditions of high perceptual load. In our experiments, we consistently failed to find any effect of perceptual load on emotional distraction. We hoped to conclude that emotional stimuli receive priority processing even in the face of high perceptual load. To do so we needed to show that non-emotional distraction is abolished by high perceptual load, as shown in many previous studies.

The basic paradigm we used is shown in Figure 1(a). A circular array of letters is presented (which we shall term the “target array”) and the participant is asked to determine which of two possible targets, either an “N” or a “Z,” was presented. At the centre of the array we could present another letter, termed the distractor, which the participant was told was irrelevant and that they should attempt to ignore. While most studies have presented the distractor at a location that is peripheral to the target array (e.g., Lavie & Cox, 1997), our studies of emotional images had placed these distractor images at the centre of the display, and so we wished to do the same for these non-emotional stimuli. Previous studies have shown that high perceptual load also serves to reduce congruence effects when the distractor is placed at the centre (Beck & Lavie, 2005)—we shall return to this issue later. Load was manipulated by using the difficulty of distinguishing the target from the non-target letters in the target array in a manner used by many other studies (e.g., Lavie & Cox, 1997). Under the low-load condition the non-targets were the letter “O,” while in the high-load the non-targets were other letters that were easily confusable with the target letter (see Figure 1).

The experiment compared the effects of four types of distractor: (a) as a baseline condition, the distractor was absent, (b) a neutral distractor (in this case an “H”) that contained similar visual properties to the other distractors, target and non-target elements, but was not part of the target set, (c) a congruent distractor—the same letter as the target letter, and (d) an incongruent distractor—the other possible target letter to the one that was present in the target array. This allowed us to examine two forms of distraction effects—a pure distraction effect, and a congruence of distractor effect.

Most studies of perceptual load examine the difference between the congruent and incongruent conditions (or sometimes the difference between an incongruent distractor and a neutral distractor). This is based on the idea that if the distractor is processed up to the stage where its representation can influence motor responses then distractors that suggest the correct response will decrease response times, while distractors that suggest the incorrect response will increase response times. We term this the “**congruence effect**.”

It has been shown that distractors that do not suggest a response can also produce a distraction effect (Jonides & Yantis, 1988). It is thought that these items demand visual processing and use up some of the processing resources and thus reduce processing of the target element. We define this “**distraction effect**” as the difference between performance when a neutral distractor is shown in comparison to when the distractor is absent. Forster and Lavie (2008) demonstrated such a distraction effect at low loads and found that the effect is reduced/abolished at high loads.

Thus, we can examine the effects of perceptual load on whether the distractor image demands attentional resources (via the distraction effect) and whether the distractor is processed to a degree where its identity can also influence performance (via the congruence effect).

## Experiment 1. Central Distractor

This was an attempted replication of the many studies that have shown that the influence of distractors is diminished/abolished under high load. We hypothesised that (a) there would be a strong effect of load, (b) there would be a distraction effect, (c) there would be a congruence effect, (d) that there would be an interaction between load and both the distraction and congruence effects, such that they will be larger at low load than at high load.

### Methods

#### Participants

The participant sample was based on a power calculation for detecting an interaction in a two-by-two analysis of variance,^1^ with a medium effects size (*F* = .25), and standard alpha (=.05) and power (=.80) and with an estimated correlation of .75 between measurements. This gave a required sample of 18. The participants were 39 (32 female, seven male) undergraduates from Cardiff University, who participated to fulfil course requirements. Thirty were aged of 18–21, eight 22–30, and one aged 41–50. Thirty-one reported their ethnicity as “White,” five as “Asian,” and four as “other.”

#### Stimuli and Procedure

Ethical permission for this and other studies reported in this article was granted by the Ethics Committee of the School of Psychology, Cardiff University. All experiments were written in Psychopy and were completed on-line via the Pavlovia website. As the study was conducted on-line, the size of displays (and the distance the person was away from the screen) will vary from participant to participant. Figures given here refer to those taken from the first author's screen with the participant sat 57 cm from the screen.

Trials consisted of a fixation cross for 300 ms, a blank screen (200 ms) and then the stimuli. These remained on the screen until a response was made. Trials were separated by a blank screen for 1,000 ms. All stimuli were black on a white background. Each stimulus consisted of a target array of four elements (see Figure 1). These elements each subtended as angle of 1° and were located 3° from the fixation point. One of the elements was a target letter than could either be a “Z” or an “N” and the location of the target element was randomised from trial to trial to be at one of the four locations. The other three possible locations in the target array were filled with an “O” for the low load condition, and with one of the letters “A,” “F,” “K,” “M,” “R,” “W,” or “Y” for the high load condition.

In addition to the target array, a distraction letter was also presented at the point of fixation (also subtending 1°). This element was either blank, a neutral letter (“H”), congruent (the same as the target letter), or incongruent (the other possible target letter to the one presented). Each of the eight conditions (two load by four distractors) had 32 trials to produce 256 trials. These trials were presented in a fresh random order for each participant. The participant completed 12 practice trials before the main block of trials. The task took around 8 min to complete.

Participant instructions stated that they had to search the circular array of possible target letters to find the target that would be a Z or an N. They were instructed to fixate the fixation point and not to move their eyes from this central location throughout the trial. They were instructed to place a finger from their left hand on the Z key and a finger from their right hand on the N key, and to press the Z or N key to indicate the target as quickly as possible while trying to avoid mistakes. They were explicitly told that they “*must try to ignore the letter in the middle*.”

#### Data Analysis

Trials on which the participant made an error were counted and removed from the analysis of reaction times (RTs). Trials on which the person responded in less than 300 ms or greater than 2,000 ms were removed. Mean RTs and percentage errors for the remaining trials in each condition were calculated. Participants who scored an overall error rate of >25% or whose overall RT was >3 SD from the mean of the other participants were removed.

Distraction and congruence effects are expected to produce poorer performance on both response rates and accuracy of task performance. However, individuals may vary in whether they slow performance to obtain a similar rate of accuracy or keep the same speed of response but at a cost of accuracy (with any combination of these two factors). To account for this, we combined the RTs and the error rate using the inverse efficiency (IE) formula which divides the mean correct RT score (for each condition and each participant) by the corresponding proportion correct (Townsend & Ashby, 1983) and these IE scores form the basis of our main analysis. IE scores are not recommended for use if error rates are high (>10%) or if there are indications of a speed-accuracy trade-off (Townsend & Ashby, 1983). We also completed more traditional analyses using the RTs and error data in separate analyses as this is recommended by some authors as a check on analyses using IE data (Bruyer & Brysbaert, 2011) and these data, along with the data relating to possible speed-accuracy trade-offs, are given in Table 1. These analyses showed the same pattern of results as the IE data and are available in the Supplemental Materials.

**Table 1.** Reaction times and error data for the six experiments.

	Distractor	Reaction times (ms)						Errors (%)						Speed-accuracy
	Load	None	Neutral	Cong	Incong	N − B	I − C	None	Neutral	Cong	Incong	N − B	I − C	*r*
Experiment 1	Low	613	670	641	751	57	111	6.1	5.5	6.2	13.3	−0.6	7.2	−.04
	High	791	851	826	920	60	95	7.2	7.3	6.3	13.5	0.1	7.2	−.10
Experiment 2	Low	524	571	555	633	47	79	4.0	5.6	4.3	20.5	1.6	15.9	−.23
	High	652	694	680	750	42	70	9.7	10.2	8.5	16.9	0.5	8.4	−.29
Experiment 3	Low	826	841	829	952	15	123	8.5	7.4	7.6	14.1	−1.1	6.5	.33
	High	1019	1089	1041	1152	70	111	9.6	9.9	8.8	12.4	0.3	3.6	.41*
Experiment 4	Low	591	604	593	624	13	31	3.5	4.6	3.6	6.8	1.1	2.8	.42*
	High	771	791	776	802	20	26	6.8	6.4	5.5	9.5	−0.4	4.0	.52*
Experiment 5	Low	542	559	556	579	17	23	7.9	6.4	5.4	11.5	−1.5	6.1	.07
	High	707	715	682	707	8	25	8.9	11.4	7.1	16.0	2.5	8.9	.35
Experiment 6	Low	667	671	676	689	4	13	6.4	5.2	6.3	7.0	−1.2	1.3	−.04
	High	890	909	886	917	19	31	8.1	9.2	7.2	8.5	1.1	1.3	.61*

The main analysis for all six experiments focused on the IE data and consisted of an omnibus two (load: high, low) by four (distractor: none, neutral, congruent, incongruent) analysis of variance (ANOVA). Degrees of freedom were corrected by the Greenhouse-Geisser formulation where Mauchly's Test of Sphericity was significant. The main analyses were followed up by calculating a distraction index (IE_neutral_ – IE_none_) and a congruence index (IE_incongruent_ – IE_congruent_). These indices were compared to zero in one-sample *t*-tests to see if distraction and congruence effects occurred, and the magnitude of these effects (e.g., low vs. high load) was compared via paired *t*-tests. * *p* <.01.

### Results and Discussion

**Figure 2.**
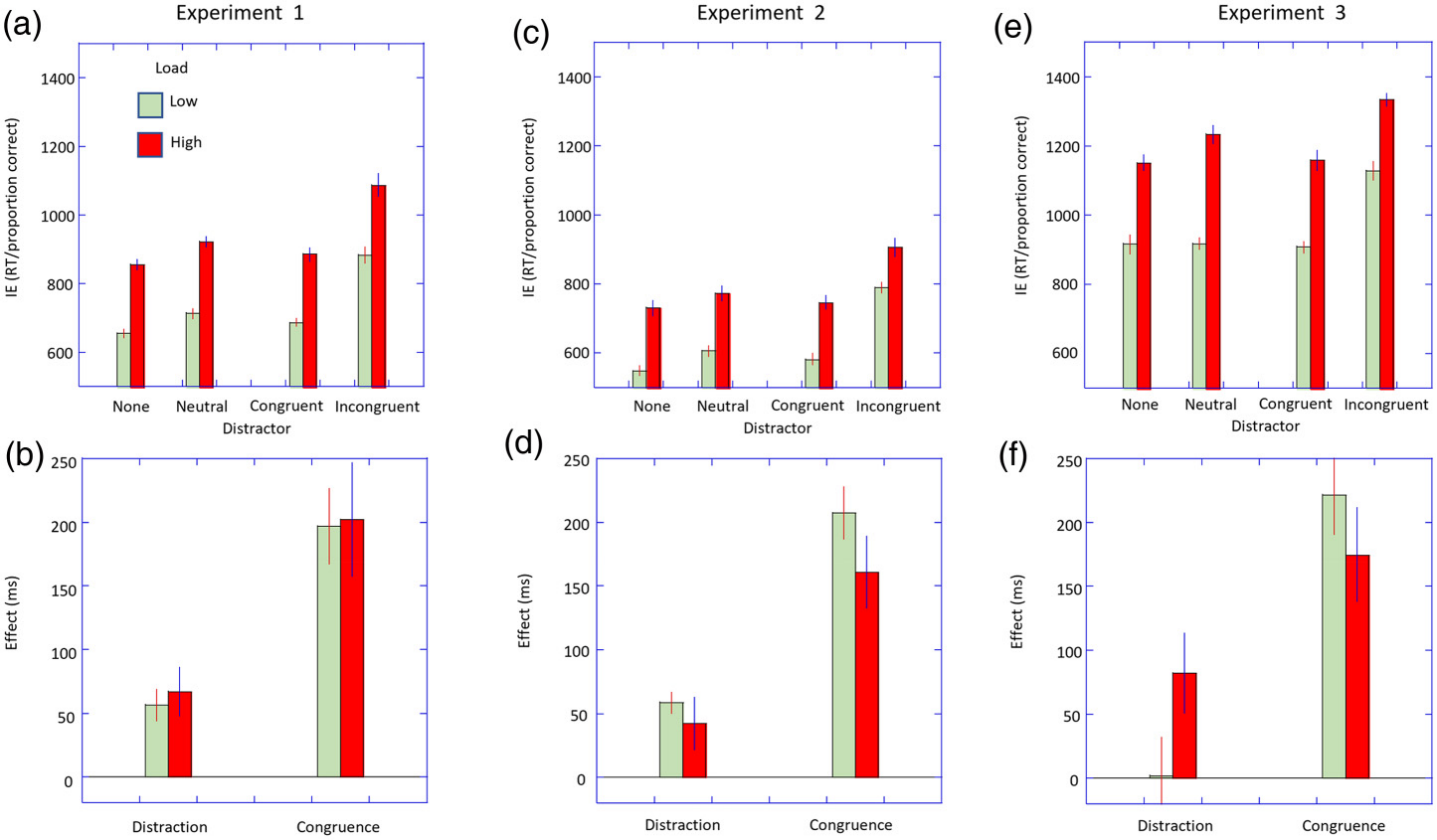
Results from Experiments 1–3. Upper panels. Error-corrected RTs (or inverse efficiency scores) are plotted as a function of distractor type stratified by the perceptual load. Error bars are ±1 within subjects SEM. Lower panels. The distraction and congruence indices are plotted stratified by the perceptual load. Error bars are ±1 SEM.

Three participants’ datasets were removed due to excessive errors. The RT and error data are given in Table 1. The IE scores for each condition and load are shown in Figure 2(a) and the distraction and congruence indices based on these are shown in Figure 2(b).

The ANOVA showed main effects of distractor type, *F*(1.5, 51.4) = 41.69, *p* < .001, η_p_^2^ = .54 and of load, *F*(1, 35) = 257.07, *p* < .001, η_p_^2^ = .88. The interaction of load and distractor type was not significant, *F*(1.8, 63.7) = 0.05, *p* = .98, η_p_^2^ < .01.

The distractibility indices were significant for both the low load (56 ms, *t*(35) = 4.79, *p* < .001, *d* = 0.80) and high load conditions (67 ms, *t*(35) = 3.58, *p* < .001, *d* = 0.60) but did not differ significantly, *t*(35) = −0.52, *p* = .60, *d* = 0.09. The congruence indices were significant for both the low load (197 ms, *t*(35) = 6.75, *p* < .001, *d* = 1.12) and high load conditions (202 ms, *t*(35) = 4.56, *p* < .001, *d* = 0.76) but did not differ significantly, *t*(35) = −0.12, *p* = .91, *d* = 0.02.

The manipulation of perceptual load was successful in producing the expected strong effects on performance. The neutral distractor produced a strong distraction effect (“large” effect sizes according to conventions; Cohen, 1988) in comparison to no distractor, even though this neutral distractor does not produce any response bias. This suggests that this distractor must attract processing resources even though it is entirely irrelevant, and the participant is instructed to ignore this element. Further, there were strong congruence effects (again with “large” effect sizes). This shows that the distractor was processed to a level where the identity of the target was realised and this in turn produced a response-bias that affected the ability to respond to the target. The crucial result was that these two effects were not influenced by the perceptual load. This clearly goes against the predictions of perceptual load theory.

## Experiment 2. Central Distractors, Brief Presentation

Most experiments showing reduced distraction at high load have used a brief presentation of the target array and distractor ostensibly to limit the opportunity of eye movements to any part of the display. Experiment 1 presented the display for an unlimited duration (until a response was made). This difference should have no effect according to perceptual load theory. Roper and Vecera (2013) added to simple load theory by suggesting there are also limits on mnemonic processes that limit encoding, which they manipulated by the duration of the displays. Under conditions of high mnemonic load (brief duration of displays) they show the classic result of a congruence effect at low, but not high, perceptual load. However, under conditions where the mnemonic load was alleviated (long duration displays) congruence effects were now also evident at high perceptual load. Roper and Vecera did not examine distraction effects. Hence, our failure to produce the classic effect of perceptual load may be due to using response terminated displays. In Experiment 2, we tested this notion by repeating Experiment 1 under the conditions of brief presentation of the all the elements of the display.

### Methods

These were the same as in Experiment 1 except that the duration of the target array and distractor which was limited to 200 ms. The participants (*N* = 32: 18 female, 14 male) were from the same pool as Experiment 1 but were not the same people (this is true for all six experiments). Twenty-nine were aged of 18–21, one 22–30, one aged 31–40, and one age 41–50. Twenty-six reported their ethnicity as “White,” three as “Asian,” two as “Black,” and one as “other.”

### Results and Discussion

No datasets were removed due to excessive errors or outlying RTs. The RT and error data are given in Table 1. The IE scores for each condition and load are shown in Figure 2(c) and the distraction and congruence indices based on these are shown in Figure 2(d).

The ANOVA showed main effects of distractor type, *F*(1.65, 51,3) = 76.54, *p* < .001, η_p_^2^ = .71 and load, *F*(1, 31) = 353.36, *p* < .001, η_p_^2^ = .92. The interaction of load and distractor type was not significant, *F*(2.6, 80.9) = 2.80, *p* = .05, η_p_^2^ = .08.

The distractibility indices were significant for both low load (58.5 ms, *t*(31) = 7.25, *p* < .001, *d* = 1.28) and high load (42.4 ms, *t*(31) = 2.34, *p* = .01, *d* = 0.41) and did not differ significantly, *t*(31) = 0.74, *p* = .23, *d* = 0.13. The congruence indices were significant for both the low load (207.5 ms, *t*(31) = 10.21, *p* < .001, *d* = 1.81) and high load (160.8 ms, *t*(31) = 5.78, *p* < .001, *d* = 1.02) and did not differ significantly, *t*(31) = 1.90, *p* = .07, *d* = 0.33.

For this experiment, there was some indication of a speed-accuracy trade-off for both the high and low load conditions (see Table 1), though this did not reach statistical significance. As a further check on whether there was an effect of load on the processing of the distractor item, we compared the congruence effect for the low and high loads using the RT data (see Table 1). These did not differ (Δ9 ms; (*t*(31) = 0.97, *p* = .34, *d* = 0.17).

Presenting the stimulus array briefly appears to have had little effect on the pattern of results. There were strong distracting effects of a neutral distractor and strong congruence effects which were of similar magnitudes across the low and high load conditions. The results once again fail to replicate the standard finding of an effect of perceptual load, but they also failed to replicate the findings of Roper and Vecera (2013). To recap, Roper and Vecera found that displays that were present until the participant responded did not show an effect of perceptual load (as in Experiment 1) on the congruence effect, but that there was an effect of perceptual load when the displays were of a brief duration (which we did not replicate in Experiment 2). We note that there are some differences between the present experiment and that of Roper and Vecera. The main difference between the present study and that of Roper and Vecera is that we chose to present all our conditions (both load and distractor type) in an interleaved set of trials, while the study of Roper and Vecera presented low load and high load trials in different blocks. This may have allowed for different attentional strategies to have been employed between the two blocks. Theeuwes et al. (2004) directly compared the effects of load under blocked conditions compared to when the trails were interleaved. For the blocked conditions there was a significant distraction effect at low loads, but not at high loads. However, when the trails were interleaved distraction effects occurred for both low and high loads and the magnitude of this distraction did not differ significantly. We believe that the interleaved conditions allow for a fairer comparison of the effects of load as this does not allow for different attentional strategies to be used in the different conditions. However, it would be of interest to reexamine the effects of stimulus duration under blocked vs interleaved trial conditions, especially given the findings that it is the duration of the target array, and not the duration of the distractor, that seems to drive the effects of stimulus duration on the perceptual load effects in the study of Roper and Vecera.

## Experiment 3. Central Distractors, Increased Load

**Figure 3.**
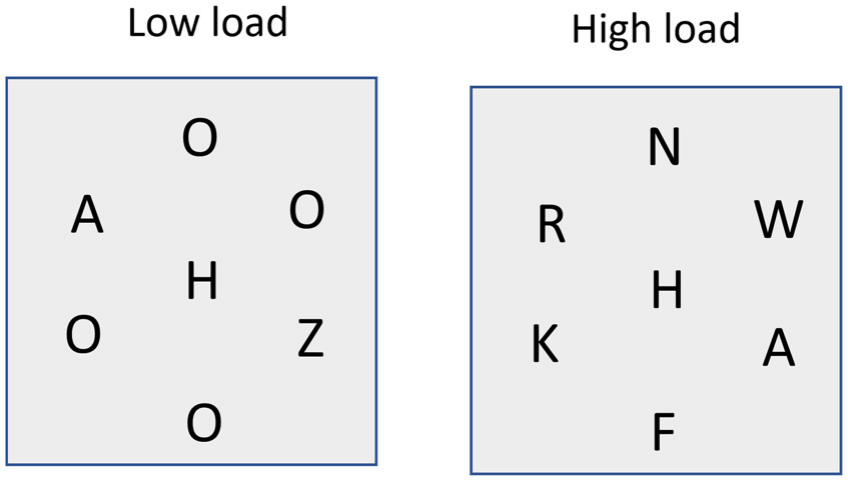
Illustration of the displays used in Experiment 3.

In Experiments 1 and 2 we used a high load of four elements. This produced a large effect in comparison to the low load (η_p_^2^ = .88 and .92). The perceptual load hypothesis states that the processing of the distractor occurs due to any processing resources that are not used up by the main target array being deployed to the distractor. Hence, we should expect to see a graded effect with less and less processing of the distractor as the load increases. However, it could be hypothesised that there is a finite amount of processing resources and if the load is below this level the distractor is processed fully, whereas if it is above this level the distractor is not processed at all. While such an idea seems implausible, especially when considered over a number of participants where there will be individual differences in any such hard limit, the results of Lavie and Cox (1997) seem to show robust congruence effects for a load of four, but this is abolished at a load of six elements. Therefore, we decided to replicate our findings under conditions of even greater load. We also took the opportunity to test a slightly different version of the low-load condition. One possible reason for the differences between low and high load conditions in most previous experiments is that in the low-load condition the target is “unique” (rather than just low load) and it is this uniqueness that allows for resources to not be required for its processing and hence distraction/congruent effects occur. Experiment 3 tested conditions under a low-load condition of two elements so that the perceptual load was still low but the target was not “unique,” and a high load condition of six elements (see Figure 3).

### Methods

These were the same as Experiment 1 save for the following changes. The target array now consisted of six elements arranged in a circle with a radius of 3° from the fixation point. The elements started directly above the fixation mark (0°) and were separated by 60° (see Figure 3). For the low load condition, the array consisted of a target (Z or N) letter and one other possible target item (randomly located with respect to the target). The other locations contained an “O.” For the high load condition, the array consisted of a target letter with the other locations containing other possible target items. The distractors were once again located at the point of fixation.

Thirty-seven (33 female, four male) people participated. Twenty-six were aged of 18–21, seven 22–30, two 31–40, and two of age 51–60. Twenty-six reported their ethnicity as “White,” eight as “Asian,” one as “Black,” and two as “other.”

### Results and Discussion

Three participant datasets were excluded due to excessive error rates. The RT and error data are given in Table 1. The IE scores for each condition and load are shown in Figure 2(e) and the distraction and congruence indices based on these are shown in Figure 2(f).

The ANOVA showed main effects of distractor type, *F*(3, 99) = 33.85, *p* < .001, η_p_^2^ = .51, and load, *F*(1, 33) = 136.29, *p* < .001, η_p_^2^ = .81. The interaction of load and distractor type was not significant, *F*(3, 99) = 2.36, *p* = .08, η_p_^2^ = .07.

The distractibility index was not significant for the low load condition (2 ms, *t*(33) = 0.07, *p* = .95, *d* = 0.01) but was for the high load condition (82 ms, *t*(33) = 2.68, *p* = .01, *d* = 0.46). However, this difference in the magnitude of the distraction effect between load failed to reach significance (*t*(33) = −1.78, *p* = .08, *d* = −0.31). The congruence indices were significant for both the low load (221 ms, *t*(33) = 7.30, *p* < .001, *d* = 1.25) and high load (175 ms, *t*(33) = 5.56, *p* < .001, *d* = 0.83) and did not differ significantly(*t*(33) = 1.06, *p* = .30, *d* = 0.18).

Once again, the results show strong effects of perceptual load and both distractor and congruence effects (though we note that the distractor effect was not significant at low load in this case). Despite the increase in the level of perceptual load from Experiment 1 there was still no effect of perceptual load on the magnitude of the distractor or congruence effects.

## Experiment 4. Peripheral Distractors

In Experiments 1–3, the location of the distractor element was at the point of fixation which differs from most other studies of perceptual load where the target array is normally placed around the point of fixation and the distractor at a peripheral location (peripheral in the sense that it was outside the target ring rather than inside it—see Beck & Lavie, 2005). We chose this central configuration for our distractor as it mimicked our studies on the effects of emotional distractors. Perceptual load theory states that attentional resources are initially used to processes the target array and only if these are not exhausted is the distractor processed. This theory does not make any distinction between centrally or peripherally displaced distractors. Nevertheless, one might imagine that elements placed at the point of fixation (centrally) may receive priority processing (though see Chen, 2008 for a different point of view). It may also benefit from a “cueing” effect due to the presentation of the fixation point at this location (Johnson et al., 2002). So, in our displays, the distractor was processed as it received this priority of resources and hence, we obtained distraction/congruence effects despite the changes in perceptual load.

The issue of central versus peripheral distractors was addressed directly by Beck and Lavie (2005). They found that central distractors produced greater congruence effects than peripheral distractors. Crucially, however, they found the same effects of perceptual load for both central peripheral distractors. At low load there were strong effects of the distractors. At high load there was no distraction effect for the peripheral distractors, while the effect of central distractors was much reduced but still significant. Hence, their results suggest that perceptual load theory holds for both central and peripheral distractors but that the strong effects of central distractors meant that there was still some distraction even at high perceptual loads. Indeed, the “strong” distraction effects produced by central distractors were the reason we chose to use this configuration in our initial experiments. The experiment of Beck and Lavie only examined congruence effects (not distraction effects).

To examine if the lack of perceptual load on distraction and congruence effects found in Experiments 1–3 was due to presenting the distractor at the point of fixation, we repeated each of these experiments but with the distractor placed at a peripheral location outside the target array ring.

### Method

In this experiment, the distractor image was to be placed in the peripheral retina outside the circumference of the target array. To accommodate this, and to make the overall size of the display equivalent to those of Experiments 1–3, the target array was reduced in circumference to 1.5°. The distractor item was placed in a parafoveal region 3° from the fixation point and could be at an angle of 45°, 135°, 225°, or 315°. The position of the distractor was manipulated between-subjects so for each participant its location was the same on all trails (to match the studies using a central target which was always at the same location).

Twenty-four participants took part (18 female, six male). Twenty were aged 18–21, three 22–30, and one 31–40. Eighteen reported their ethnicity as “White,” one as “Asian,” two as “Black,” and three as “other.”

### Results and Discussion

**Figure 4.**
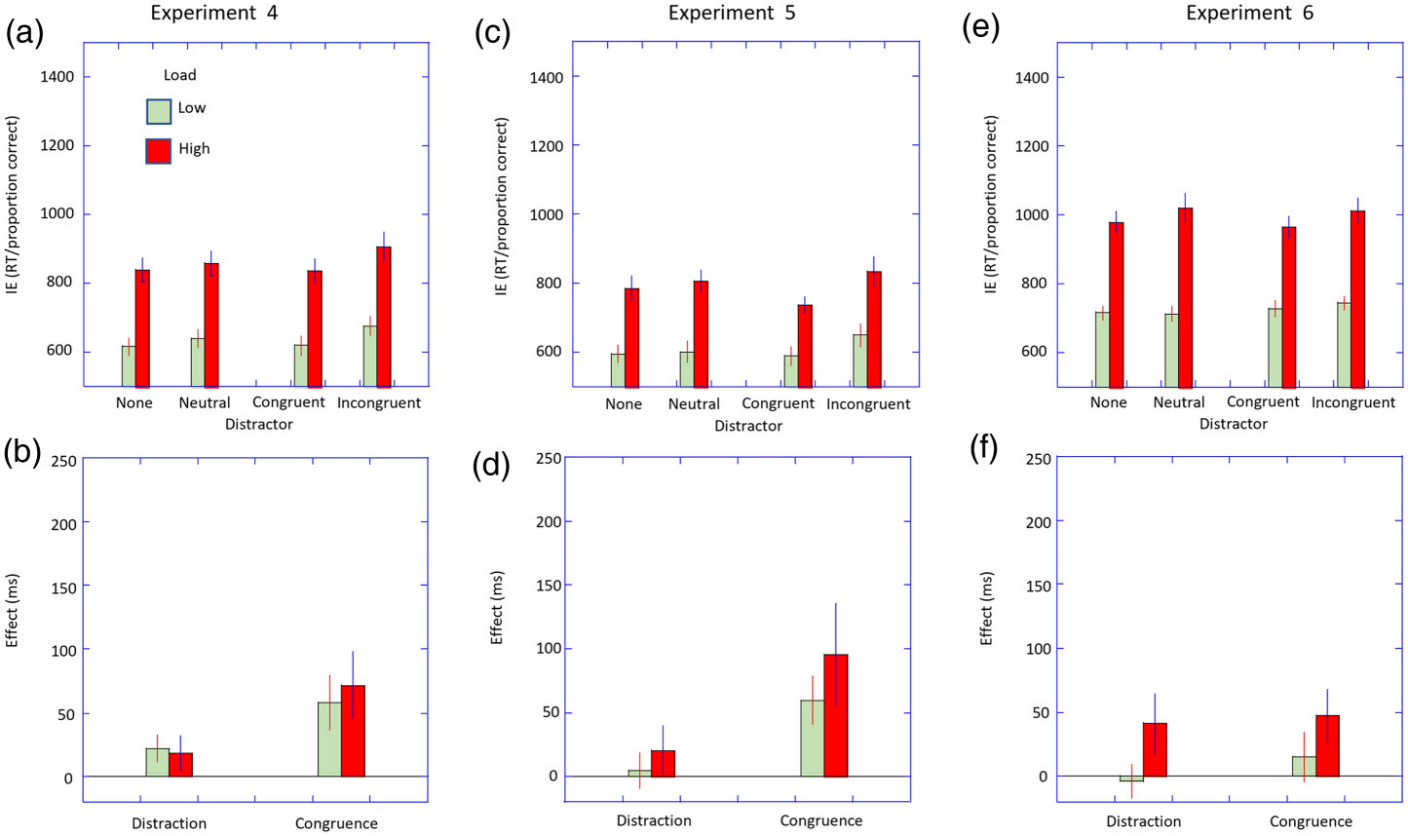
Results from Experiments 4–6. Upper panels. Error-corrected RTs (or inverse efficiency scores) are plotted as a function of distractor type stratified by the perceptual load. Error bars are ±1 within subjects SEM. Lower panels. The distraction and congruence indices are plotted stratified by the perceptual load. Error bars are ±1 SEM.

No datasets were removed due to excessive errors or outlying RTs. The RT and error data are given in Table 1. The IE scores for each condition and load are shown in Figure 4(a) and the distraction and congruence indices based on these are shown in Figure 4(b).

The ANOVA showed main effects of distractor type, *F*(1.9, 44.0) = 9.84, *p* < .001, η_p_^2^ = .30, and load, *F*(1, 23) = 82.33, *p* < .001, η_p_^2^ = .78. The interaction of load and distractor type was not significant, *F*(2.5, 58.0) = 0.13, *p* = .94, η_p_^2^ < .01.

The distractibility index was significant for the low load condition (22 ms, *t*(23) = 2.22, *p* = .04, *d* = 0.45) but was not significant for the high load condition (19 ms, *t*(23) = 1.40, *p* = .17, *d* = 0.29). However, this difference in the magnitude of the distraction effect between loads was not significant, *t*(23) = 0.24, *p* = .82, *d* = 0.05. The congruence indices were significant at low load (58 ms, *t*(23) = 2.76, *p* = .01, *d* = 0.56) and high load (72 ms, *t*(23) = 2.78 *p* = .01, *d* = 0.57) and did not differ significantly, *t*(23) = −0.50, *p* = .62, *d* = 0.10.

The experiment showed a strong effect of perceptual load as expected confirming this manipulation was successful. In line with the results of Beck and Lavie (2005), the magnitude of the congruence effect appears considerably smaller than that obtained when the distractor was central (see Figure 2), and we extend this to show that this applies to the distraction effect as well. The crucial result was that the magnitude of these distraction and congruence effects was similar in size for both low and high perceptual loads. The results are against the predictions of perceptual load theory which argues that the effects of the distractor would reduce at high perceptual loads.

## Experiment 5. Peripheral Distractors, Brief Presentation

### Methods

These were the same as in Experiment 4 except that the duration of the target array and distractor was limited to 200 ms. Nineteen participants took part (13 female, five male, one other). Fourteen were aged 18–21, four 22–30, and one aged 31–40. Fifteen reported their ethnicity as “White,” three as “Asian,” and one as “other.”

### Results and Discussion

One dataset was removed due to excessive error. The RT and error data are given in Table 1. The IE scores for each condition and load are shown in Figure 4(c) and the distraction and congruence indices based on these are shown in Figure 4(d).

There were main effects of distractor type, *F*(1.7, 29.4) = 5.92, *p* = .002, η_p_^2^ = .26, and load, *F*(1, 17) = 150.62, *p* < .001, η_p_^2^ = .90. The interaction of load and distractor type was not significant, *F*(2.4, 40.2) = 1.51, *p* = .22, η_p_^2^ = .08.

The distractibility index was not significant for the low load (5 ms, *t*(17) = 0.36, *p* = .36, *d* = 0.08) nor for the high load (21 ms, *t*(17) = 1.12, *p* = .28, *d* = 0.27) and these did not differ significantly, *t*(17) = −0.77, *p* = .45, *d* = 0.18. The congruence indices were significant for both low load (60 ms, *t*(17) = 3.28, *p* = .004, *d* = 0.77) and high load (95 ms, *t*(17) = 2.43, *p* = .03, *d* = 0.57) but these did not differ significantly, *t*(17) = −1.06, *p* = .31, *d* = 0.25.

The results showed the expected effects of perceptual load and distractor type. Against the predictions of load theory there was no effect of perceptual load on either the distractor or congruence effects.

## Experiment 6. Peripheral Distractors, Increased Load

### Methods

These were the same as Experiment 3, save that the target array had a radius of 1.5° and the distractor was placed 3° in the periphery (as in Experiments 4 and 5). Thirty-four participants took part (29 female, five male). Thirty were aged 18–21, three 22–30, and one 41–50. Twenty-nine reported their ethnicity as “White,” one as “Asian,” one as “Black,” and three as “other.”

### Results and Discussion

One participant's dataset was removed due to excessive errors and one due to long RTs. The RT and error data are given in Table 1. The IE scores for each condition and load are shown in Figure 4(e) and the distraction and congruence indices based on these are shown in Figure 4(f).

The ANOVA showed that the main effect of distractor type failed to reach significance, *F*(3, 93) = 2.32, *p* = .08, η_p_^2^ = .07, but there was a main effect of load, *F*(1, 31) = 150.22, *p* < .001, η_p_^2^ = .83. The interaction of load and distractor type was marginally significant, *F*(3, 93) = 2.75, *p* = .047, η_p_^2^ = .08.

The distractibility index was not significant for the low load condition (−3 ms, *t*(31) = −0.26, *p* = .80, *d* = 0.05) nor for the high load condition (41 ms, *t*(31) = 1.78, *p* = .09, *d* = 0.31). The difference was not significant (*t*(31) = −1.92, *p* = .06, *d* = 0.34). The congruence index was not significant for the low load (15 ms, *t*(31) = 0.81, *p* = .42, *d* = 0.24) but was for the high load (47 ms, *t*(31) = −2.31, *p* = .03, *d* = 0.41) though these indices did not differ significantly (*t*(31) = 1.16, *p* = .26, *d* = 0.21). The results again show that distraction and congruence effects are not reduced by the increasing perceptual load.

## General Discussion

Across a series of six experiments, we found:

1. increases in perceptual load increase the amount of time taken to respond to the target element;
2. a neutral distractor element that is presented at a different location to the target array produces a distraction effect in comparison to no such distractor. These results occurred even though the position of the distractor was consistent and known to the participant in all trials, and that instructions were given to ignore this item. We note, however, this effect appeared less reliable when the distractor was presented more peripheral to the target array;
3. the identity of the distractor caused a congruence effect;
4. these distractor and congruence effects are not altered by the perceptual load, and
5. the pattern of results was replicated if we used the more conventional measure of RTs.

Clearly, these results do not support perceptual load theory that states that the processing of the distractor item (and therefore the distractor and congruence effects) is dependent on the perceptual load, so that distraction can occur at low load but is reduced/abolished by high loads. The results also appear to contradict the large number of empirical findings that show such a reduction in distraction/congruence effects at high loads (see Murphy et al., 2016).

We should also add that the present results are not in accord with most alternatives to perceptual load theory. For instance, dilution theory (Tsal & Benoni, 2010) suggests that the increased number of possible targets (ones that are similar in simple features) in the target array is responsible for diluting the effects of the distractor. We did not find any change in distraction with increased number of such possible targets. Others (Neokleous et al., 2016) have used the idea of “salience” maps to model the reduction in the distraction effect with increased perceptual load (though these models do not use the concept of perceptual load to explain the results)—see also Scalf et al. (2013). Again, our finding that there is no such effect of perceptual load is at odds with such models.

There are, however, a few findings that appear to offer some possible support for the idea that the congruence is not affected by load (Yeshurun & Marciano, 2013) and a report that the distraction effect is also obtained in most high load conditions (Lleras et al., 2017). As mentioned earlier, Theeuwes et al. (2004) also found distraction effects under high perceptual load under conditions where the trials with high and low loads were interleaved but not when they were blocked. We agree with Theeuwes et al. that using a blocked design might allow participants to develop different strategies for the low and high load conditions and is therefore not a proper test of the effects of perceptual load *per se*. A second aspect of the results of Theeuwes et al. is that they show in the interleaved condition that the nature of the previous trial affects the level of distraction on the current trial. Specifically, when the current trial was of high load distraction effects were still seen if the previous trial had been low load but was reduced if the previous trial was high load. Eayrs et al. (2024) have extended these results to show that under conditions where the load of the current trial is cued, the cueing per se does not influence the magnitude of distraction. However, under the specific condition where a high load is cued and the previous trial was also high load, the levels of distraction was greatly reduced.

The differences in distraction across blocked vs interleaved trials, and the effects of previous trial, are thought to be indicative of changes in the breadth of attention (Theeuwes et al., 2004). Under some conditions, such as when all the low load trials are blocked, attention is spread widely and the distractor is processed. However, under some conditions, blocked high load trials, attention may be “narrowed” to the location of the targets and the distractor is excluded and produces little distraction. When low and high load trials are intermixed, no such narrowing of attention occurs and distraction is seen under both low and high load conditions (see also Biggs & Gibson, 2018; Cave & Chen, 2016; Chen & Cave, 2016; Eayrs et al., 2024).

Cosman and Vecera (2012) also showed distraction effects at high load under conditions where the target and distractors were grouped as a single object, but not when they were seen as different objects (though we note that under the different object conditions there were no distraction effects for low load either). A conceptually similar set of results was found by Biggs and Gibson (2018). They found that demarcating the area of the target with a circle reduced the effect of a distracting item placed outside this circle and this occurred at both low and high perceptual loads.

All these results suggest that the ability to segregate and limit attention to specific items is fundamental to whether the distraction element is processed (and therefore causes distraction and/or congruence effects), with load merely being a manipulation which may aid such segregation under some circumstances. However, such speculations do not seem to explain the discrepency between the present findings and the “classic” finding of reduced distractor processing at high loads. It is possible that there is something about the displays used in the present experiments that caused such a perceptual grouping so that the observer was always forced to process the distractor, while such a grouping was not present in other experiments. However, to our eye, the displays we used were highly similar to the canonical displays previously used (e.g., Lavie & Cox, 1997). Manipulations of factors that might group or segregate the target and distractor arrays may be informative and we hope to report on such experiments soon. It is also possible that other factors, such as the duration of the display, may also be important (Roper & Vecera, 2013) in producing exclusion of the distractor item from processing, and that the manipulation of duration in the present studies were not sufficient to produce this effect.

The experiments reported here present the data at the group, or aggregate, level. Others have suggested that there may be quite dramatic individual differences in the pattern of results, with some people demonstrating results consistent with perceptual load theory (i.e., a reduction/elimination of congruence effects at high load), and others showing results that are not consistent (Fitousi & Wenger, 2011), or even the opposite (i.e., greater distraction at high perceptual load: Marciano & Yeshurun, 2017). The study of Eayrs and Lavie (2021) suggests that individual differences in levels of subitising may influence at what load people are able still process the distractor as load increases. In their study, distraction was apparent at low loads (approximately 1–4 set size) but was not present at high loads with this “transition load” being dependent on subitising capacity. We also note that individual differences in working memory capacity have been shown to affect levels of distraction, though this appears to be a greater influence for low load conditions with its effects diminished or abolished for high loads (for a review see De Fockert, 2013). In the present studies we did not take any measures of individual differences. However, we present the results at the level of the individuals in the Supplemental Materials. Inspection of these results shows that the patterns of the results reported in this article at the aggregate (group) level are mirrored by most individuals. While there are occasional individual results that appear to run contrary to the aggregate results, these did not appear to occur in any systematic way or in any particular condition.

In summary, we have failed to replicate, over a wide range of conditions, the standard finding that distraction effects are reduced/eliminated under conditions of high perceptual load. These results show that load theory is, at best, an incomplete explanation of how stimuli are selected, and the fate of the non-attended stimuli. These results, and others reviewed in this Discussion, suggest that this theory is not yet in a position to be used in applied settings (Lleras et al., 2017). They also suggest that great caution is exercised in the interpretation of investigations using this paradigm in such areas as the processing of emotional stimuli (Yates et al., 2010), the effects of such individual difference variables such as age (Maylor & Lavie, 1998), or clinical presentations (Forster & Lavie, 2016; Sadeh & Bredemeier, 2011). Further work is needed to understand the conditions under which distraction and/or congruence effects are manifest and under what conditions perceptual load theory is appropriate so that the paradigm can be applied with a greater understanding of the psychological processes at work.

## Supplemental Material

sj-docx-1-pec-10.1177_03010066251364203 - Supplemental material for Reduction in distraction due to perceptual load: A failure to replicate Supplemental material, sj-docx-1-pec-10.1177_03010066251364203 for Reduction in distraction due to perceptual load: A failure to replicate by Robert J. Snowden and Nicola S. Gray in Perception

## Notes

1. The overall analysis was two (load) by four (distractor types) which would have required a sample size of 14, but we intended to look at the distraction effect (none, neutral) and the congruence effect (congruent, incongruent) separately and so powered the experiment for this.
