# Supplementary material for: Reduction in distraction due to perceptual load: A failure to replicate
Source: Perception. 2025 Aug 6;54(12):931–47. doi: 10.1177/03010066251364203 (PMC12605277; doi:10.1177/03010066251364203)

Supplemental Table 1. Results from ANOVA on reaction time data for Experiments 1- 6.

|  |  | All distractors | | | |  | None vs neutral | | | |  | Congruent vs incongruent | | | |
| --- | --- | --- | --- | --- | --- | --- | --- | --- | --- | --- | --- | --- | --- | --- | --- |
|  |  | *F* | *df* | *p* | *η_p_^2^* |  | *F* | *df* | *p* | *η_p_^2^* |  | *F* | *df* | *p* | *η_p_^2^* |
|  |  |  |  |  |  |  |  |  |  |  |  |  |  |  |  |
| Expt 1 | Distractors | 103.51 | 3, 105 | < .001 | .74 |  | 70.00 | 1, 35 | <.001 | .64 |  | 129.15 | 1, 35 | <.001 | .79 |
|  | Load | 316.92 | 1, 35 | < .001 | .90 |  | 302.54 | 1, 35 | <.001 | .90 |  | 213.45 | 1, 35 | <.001 | .86 |
|  | Interaction | 0.38 | 3, 105 | .38 | .01 |  | 0.04 | 1, 35 | .85 | .00 |  | 0.77 | 1, 35 | .39 | .02 |
|  |  |  |  |  |  |  |  |  |  |  |  |  |  |  |  |
| Expt 2 | Distractors | 79.72 | 3, 93 | < .001 | .72 |  | 86.91 | 1, 31 | <.001 | .74 |  | 70.18 | 1, 31 | <.001 | .69 |
|  | Load | 371.56 | 1, 31 | < .001 | .92 |  | 220.63 | 1, 31 | <.001 | .88 |  | 341.65 | 1, 31 | <.001 | .92 |
|  | Interaction | 0.42 | 3, 93 | .74 | .01 |  | 0.19 | 1, 31 | .67 | .01 |  | 0.94 | 1, 31 | .34 | .03 |
|  |  |  |  |  |  |  |  |  |  |  |  |  |  |  |  |
| Expt 3 | Distractors | 26.30 | 3, 99 | < .001 | .44 |  | 7.35 | 1, 33 | .01 | .18 |  | 86.52 | 1, 33 | <.001 | .72 |
|  | Load | 182.05 | 1, 33 | < .001 | .85 |  | 132.02 | 1, 33 | <.001 | .80 |  | 142.04 | 1, 33 | <.001 | .81 |
|  | Interaction | 1.48 | 3, 99 | .23 | .04 |  | 3.45 | 1, 33 | .07 | .10 |  | 0.23 | 1, 33 | .63 | .01 |
|  |  |  |  |  |  |  |  |  |  |  |  |  |  |  |  |
| Expt 4 | Distractors | 9.22 | 3, 69 | < .001 | .29 |  | 7.47 | 1, 23 | .01 | .25 |  | 17.08 | 1, 23 | <.001 | .43 |
|  | Load | 154.93 | 1, 23 | < .001 | .87 |  | 122.33 | 1, 23 | <.001 | .84 |  | 127.93 | 1, 23 | <.001 | .85 |
|  | Interaction | 0.13 | 3, 69 | .94 | .01 |  | 0.27 | 1, 23 | .61 | .01 |  | 0.12 | 1, 23 | .74 | .01 |
|  |  |  |  |  |  |  |  |  |  |  |  |  |  |  |  |
| Expt 5 | Distractors | 3.23 | 3, 51 | .03 | .16 |  | 2.10 | 1, 17 | .17 | .11 |  | 7.34 | 1, 17 | .02 | .30 |
|  | Load | 129.30 | 1, 17 | < .001 | .88 |  | 99.78 | 1, 17 | <.001 | .85 |  | 105.09 | 1, 17 | <.001 | .86 |
|  | Interaction | 2.84 | 3, 51 | .05 | .14 |  | 0.49 | 1, 17 | .49 | .03 |  | 0.01 | 1, 17 | .93 | .00 |
|  |  |  |  |  |  |  |  |  |  |  |  |  |  |  |  |
| Expt 6 | Distractors | 2.28 | 3, 93 | .09 | .07 |  | 0.93 | 1, 31 | .34 | .03 |  | 4.00 | 1, 31 | .05 | .11 |
|  | Load | 396.69 | 1, 31 | < .001 | .93 |  | 353.36 | 1, 31 | <.001 | .92 |  | 275.79 | 1, 31 | <.001 | .90 |
|  | Interaction | 1.10 | 3, 93 | .35 | .03 |  | 0.80 | 1, 31 | .38 | .03 |  | 1.67 | 1, 31 | .21 | .05 |
|  |  |  |  |  |  |  |  |  |  |  |  |  |  |  |  |

Supplemental Figure 1. Results from Experiment 1.


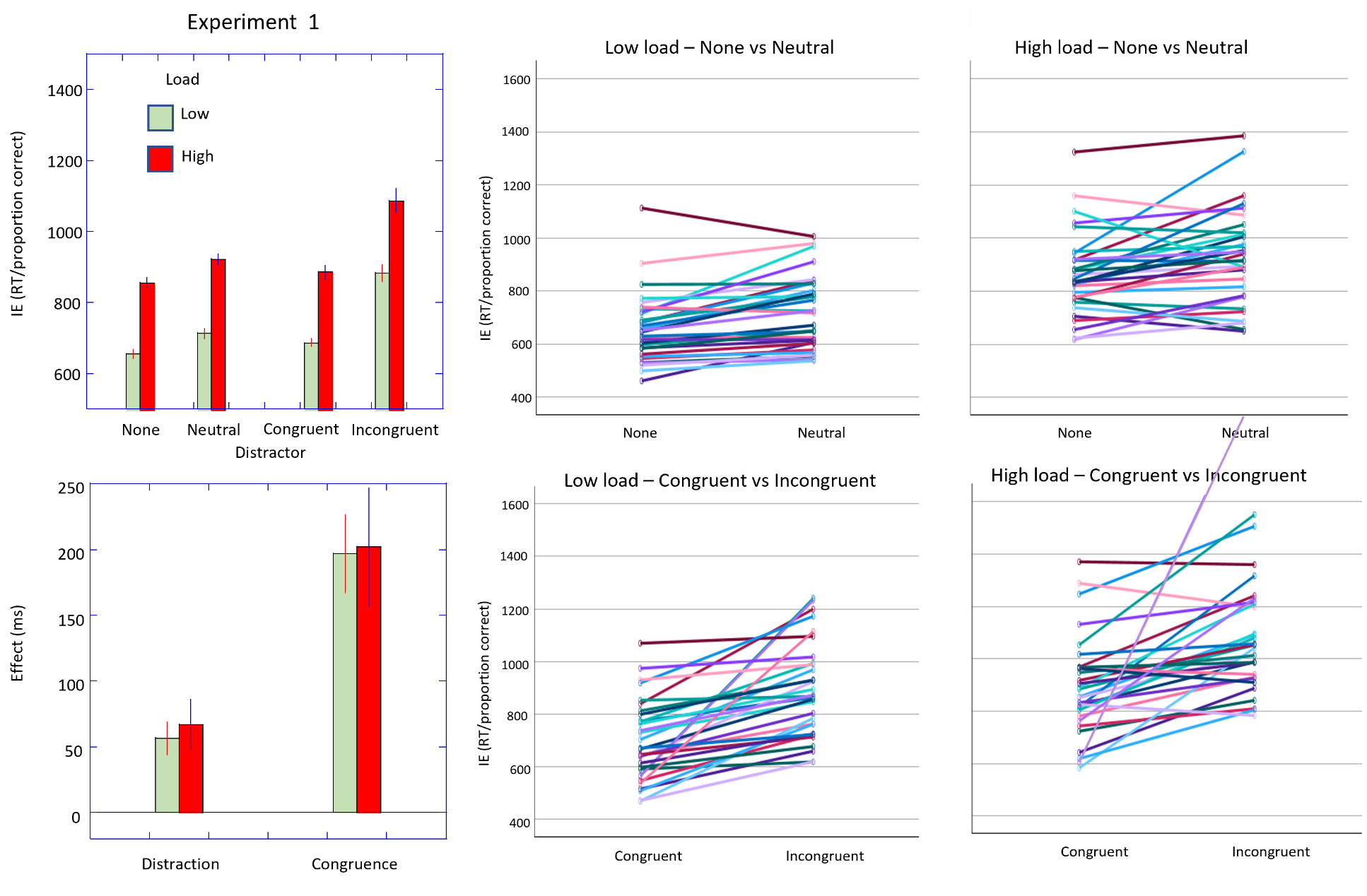


Supplemental Figure 2. Results from Experiment 2
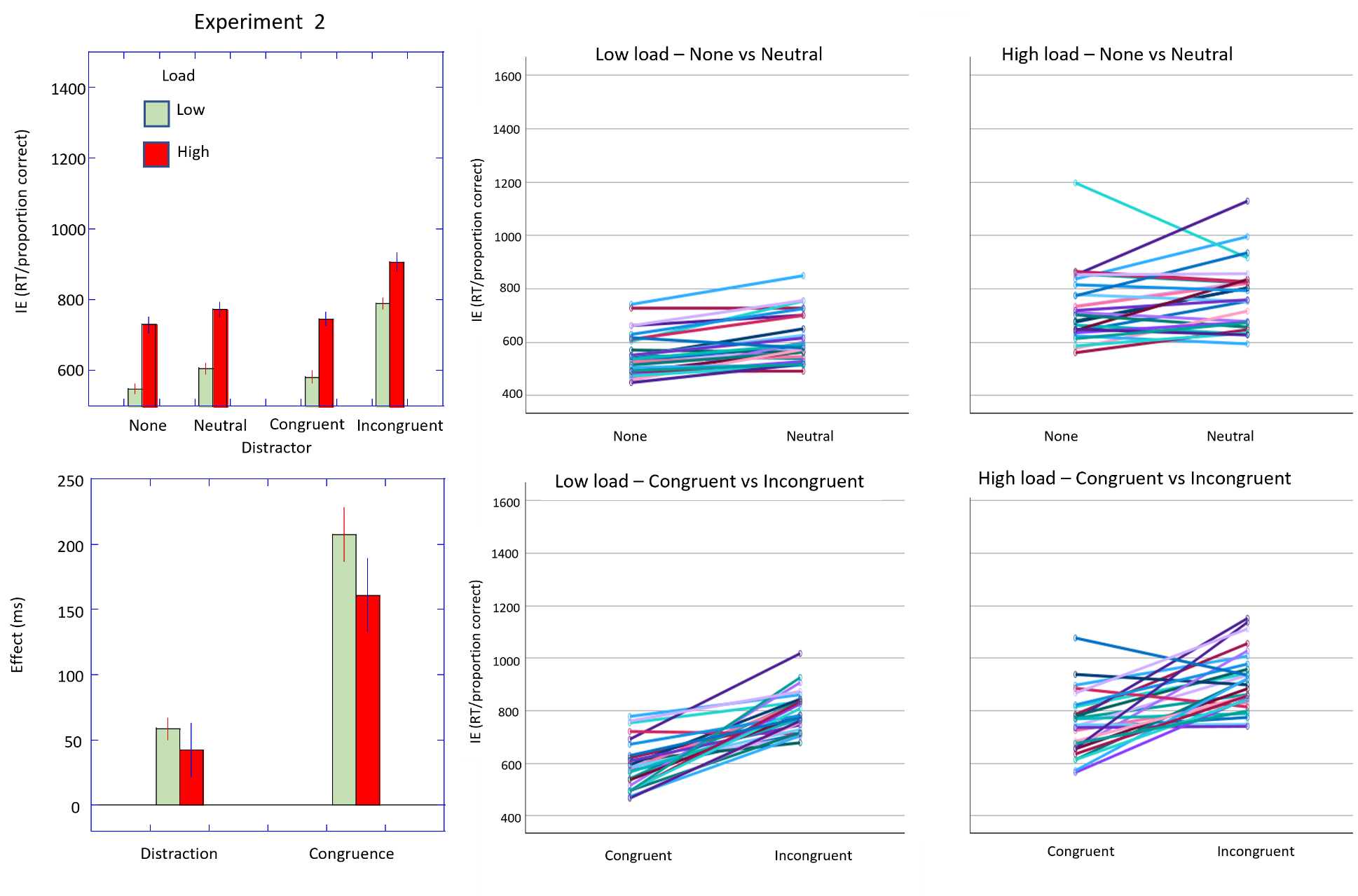
.

Supplemental Figure 3. Results from Experiment 3.


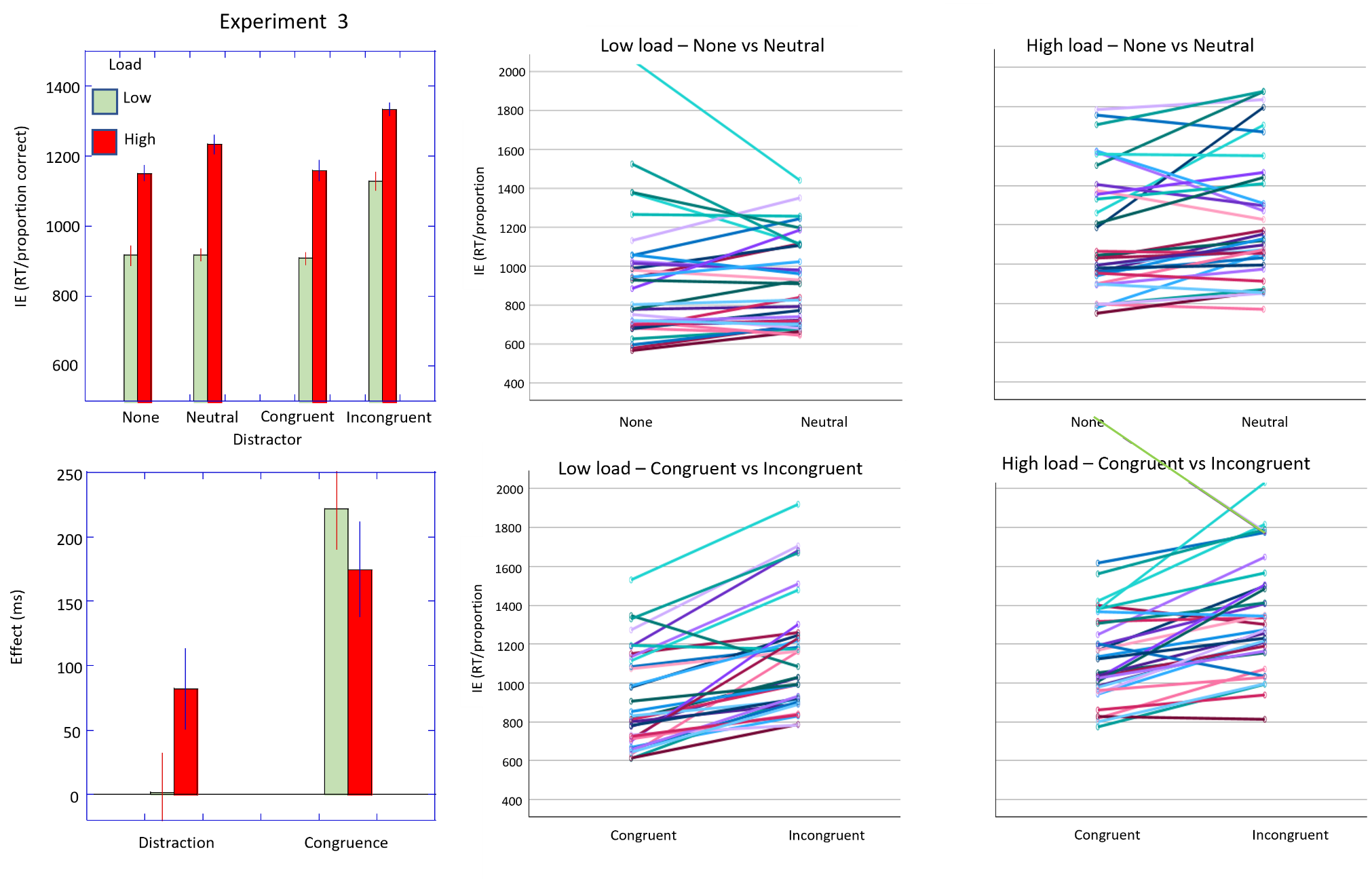


Supplemental Figure 4. Results from Experiment 4.


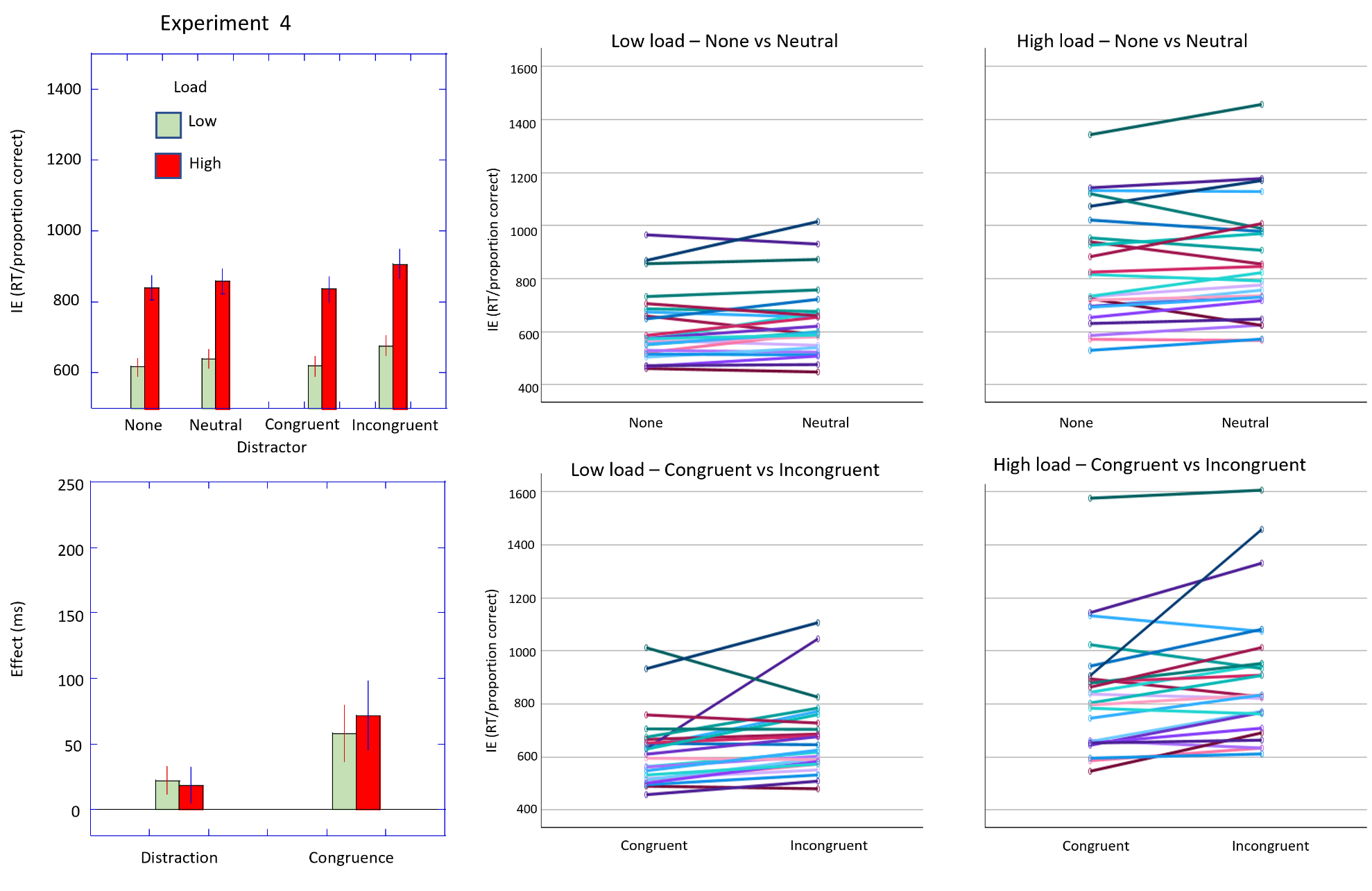


Supplemental Figure 5. Results from Experiment 5.


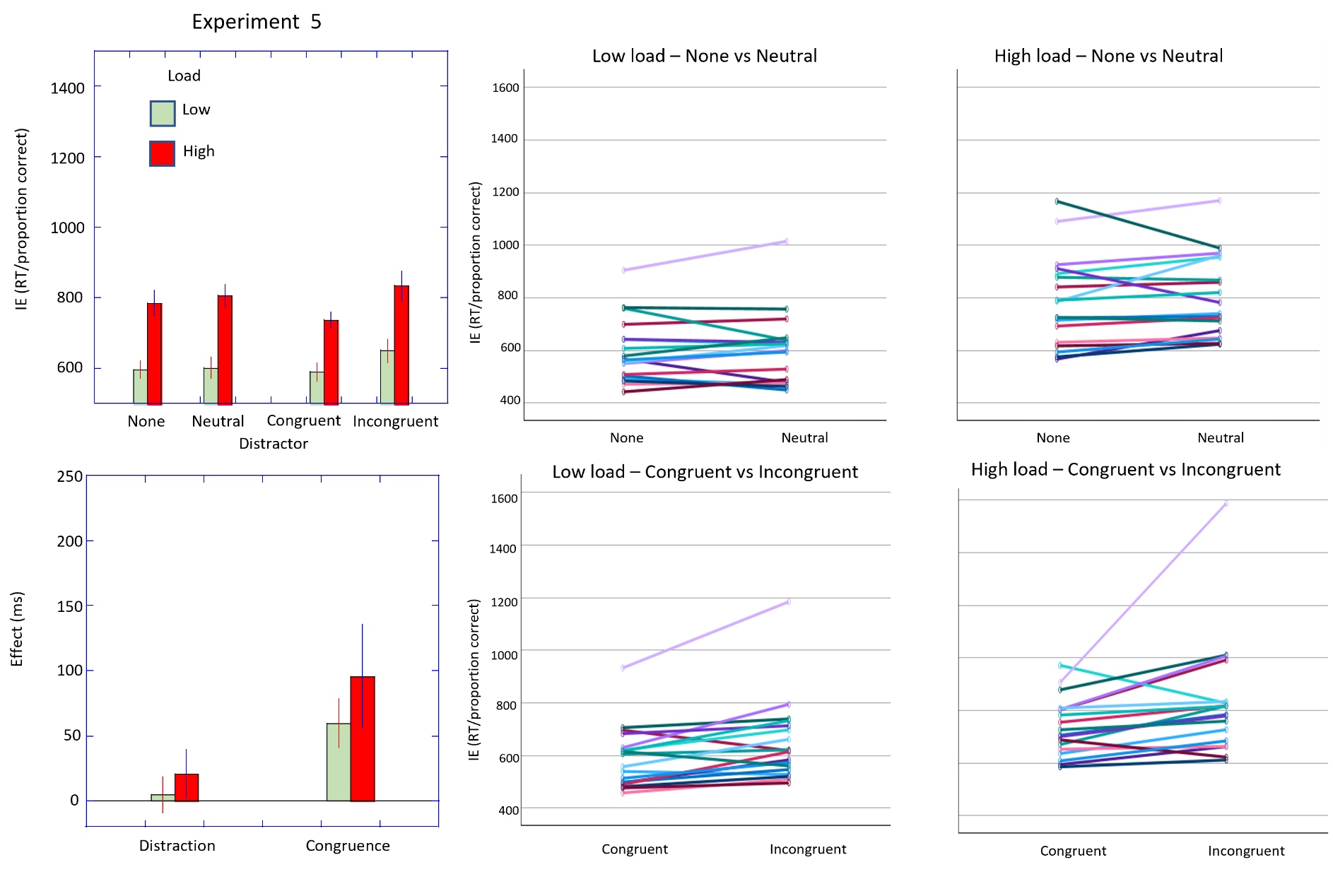


Supplemental Figure 6. Results from Experiment 6.


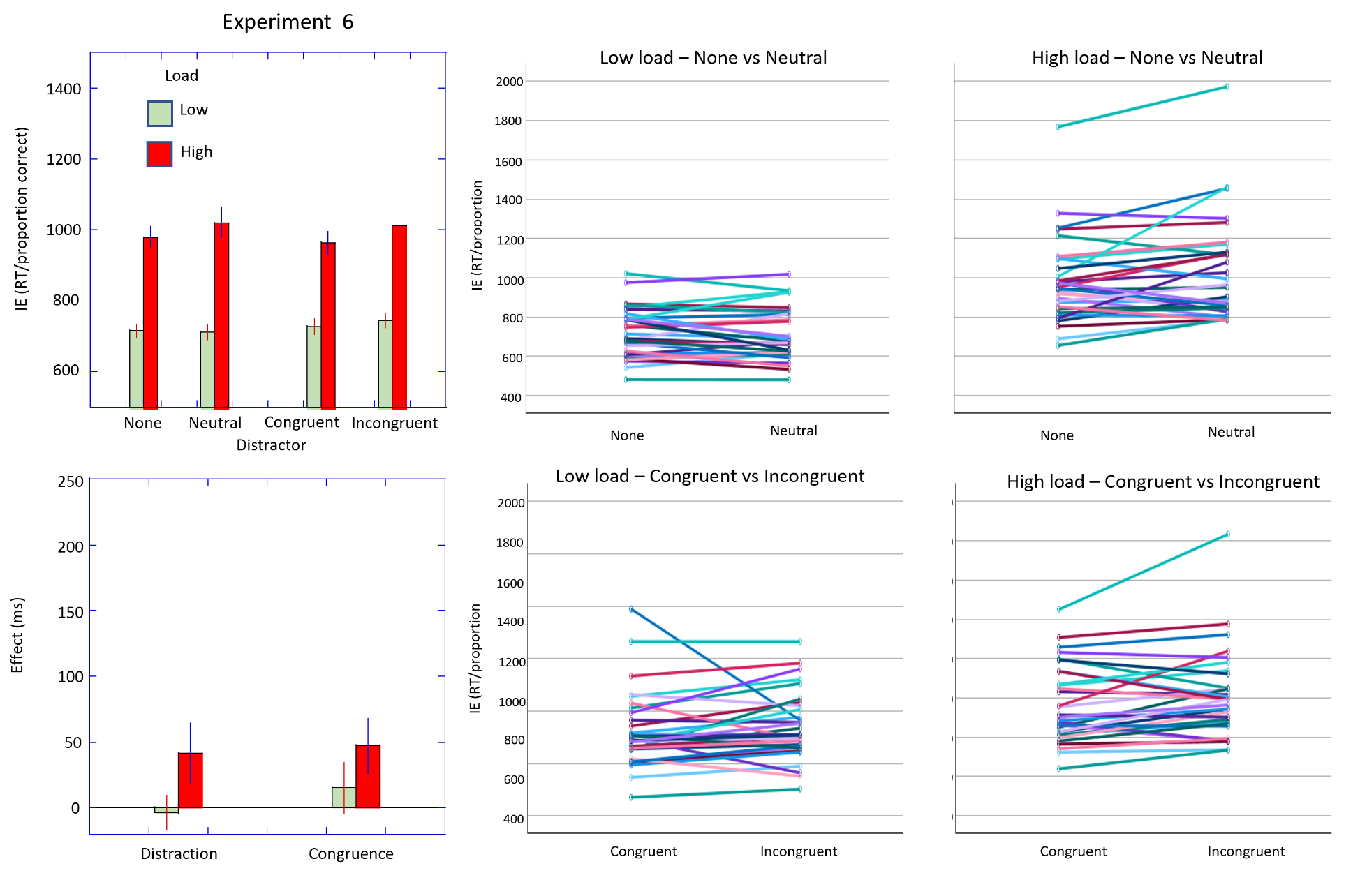

Supplement: sj-docx-1-pec-10.1177_03010066251364203 - Supplemental material for Reduction in distraction due to perceptual load: A failure to replicate [file sj-docx-1-pec-10.1177_03010066251364203.docx]
